# An Investigation on Platelet Transport during Thrombus Formation at Micro-Scale Stenosis

**DOI:** 10.1371/journal.pone.0074123

**Published:** 2013-10-23

**Authors:** Francisco Javier Tovar-Lopez, Gary Rosengarten, Mahyar Nasabi, Vijay Sivan, Khashayar Khoshmanesh, Shaun P. Jackson, Arnan Mitchell, Warwick S. Nesbitt

**Affiliations:** 1 Microplatforms Research Group, School of Electrical and Computer Engineering, Royal Melbourne Institute of Technology University, Melbourne, Victoria, Australia; 2 School of Aerospace, Mechanical and Manufacturing Engineering, Royal Melbourne Institute of Technology University, Melbourne, Victoria, Australia; 3 Department of Mechanical Engineering, Stanford University, Stanford, California, United States of America; 4 The Australian Centre for Blood Diseases, Monash University, Alfred Medical Research and Educational Precinct, Melbourne, Victoria, Australia; 5 The Bionics Institute, Melbourne, Victoria, Australia; University of Leuven, Belgium

## Abstract

This paper reports on an investigation of mass transport of blood cells at micro-scale stenosis where local strain-rate micro-gradients trigger platelet aggregation. Using a microfluidic flow focusing platform we investigate the blood flow streams that principally contribute to platelet aggregation under shear micro-gradient conditions. We demonstrate that relatively thin surface streams located at the channel wall are the primary contributor of platelets to the developing aggregate under shear gradient conditions. Furthermore we delineate a role for red blood cell hydrodynamic lift forces in driving enhanced advection of platelets to the stenosis wall and surface of developing aggregates. We show that this novel microfluidic platform can be effectively used to study the role of mass transport phenomena driving platelet recruitment and aggregate formation and believe that this approach will lead to a greater understanding of the mechanisms underlying shear-gradient dependent discoid platelet aggregation in the context of cardiovascular diseases such as acute coronary syndromes and ischemic stroke.

## Introduction

**Figure 1:**
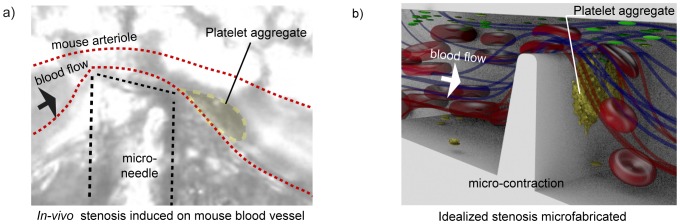
Left: A representative mouse mesenteric arteriole undergoing compression with a glass microneedle (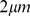 in diameter). Platelet aggregation (shown in yellow) in the absence of soluble platelet agonists signalling (ADP, thromboxane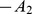 & thrombin) is found as a function of the shear gradient [*5*]. *Right: A micro contraction with an idealized geometry to maximize platelet response is microfabricated to mimic the hemodynamics conditions of the induced stenosis in the in-vivo experiment* [*4]*, [*5*] *.*

**Figure 2:**
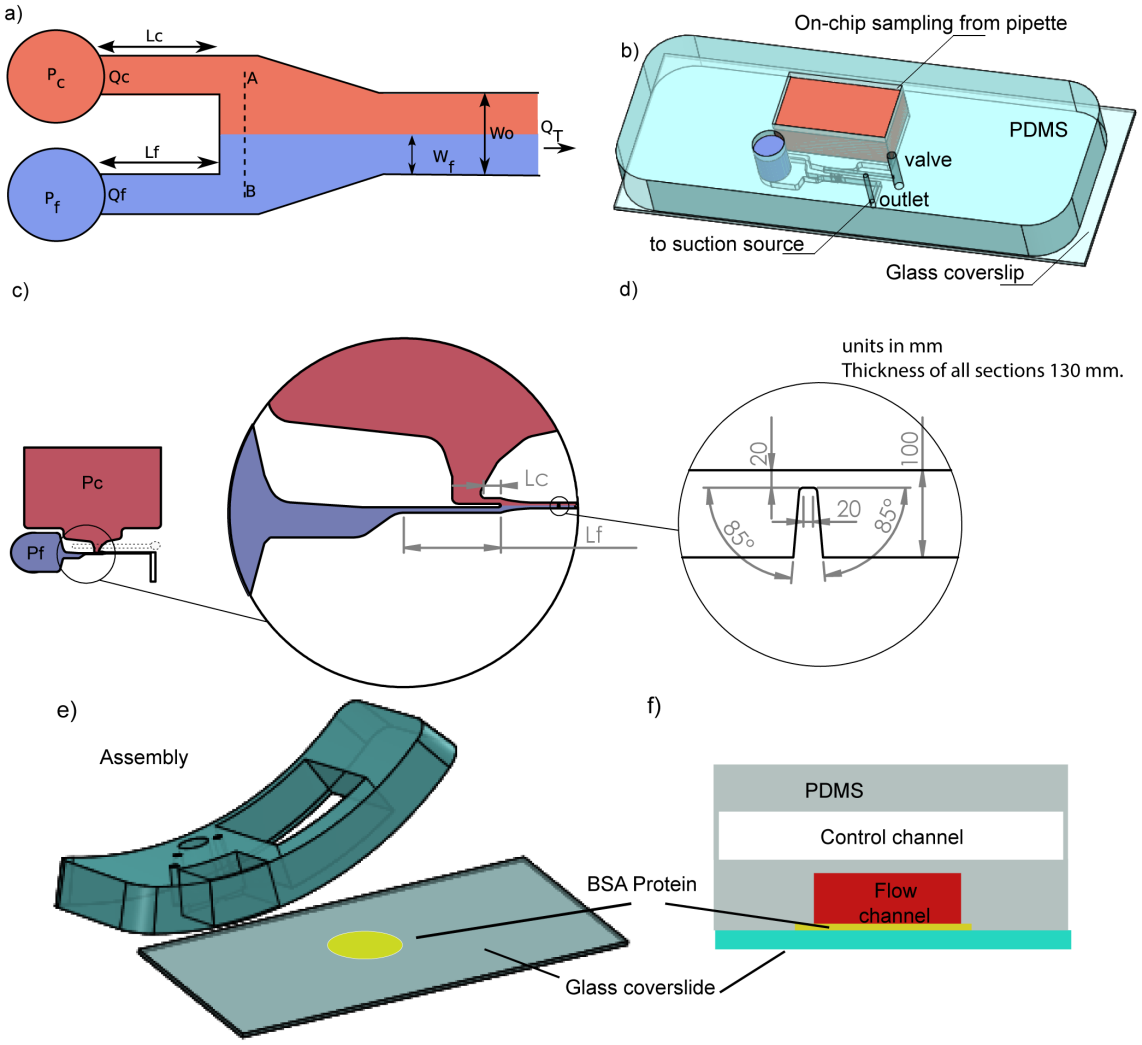
A microfluidics flow focusing device was used to investigate the role of mass transport in micro stenosis. *The device was designed to operate with negative pressure (on-chip sampling), using a single syringe pump. a) Schematic of the variables used to determine the thickness of the blood streams. Different widths of the focused stream () was achieved by changing the hydraulic resistance of the inlet feeder channels. See (Text S1). b) A 3D representation of a device fabricated in PDMS-glass (cover-slip of* 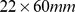 *). c) Detail of the sampling section. Pc, Pf are the reservoirs from the focused and core stream, Lc and Lf the variables to modulate the thickness of the stream. See (Text S1). d) Detail of the microcontraction (stenosis). e) Assembly of the device, showing that Bovine Serum Albumin was used to prevent unspecific adhesion of platelets to the glass surface. f) Schematic of the implemented valve to assist the purging of the device.*

Pathological thrombus formation underlies a number of major health problems with significant economic impact. Usually thrombus formation occurs where blood vessels become narrowed (stenosis) as a result of atherosclerosis. This narrowing produces changes in blood-flow parameters which in turn trigger cell adhesion and aggregation. The way in which the geometry of the blood vessel changes blood-flow parameters and these in turn affect blood cell responses has been the focus for decades. Clinically, however it has been difficult to investigate, under controlled conditions, the role of mechanical parameters (geometry and flow) on thrombosis and platelet aggregation. The challenge arises from the fact that the geometrical and flow parameters, are difficult to isolate in-vivo. Recent clinical studies still continue to neglect the geometry of the stenosis [1]–[3], concentrating just on the degree of the occlusion, neglecting the shape of the contraction, even though it has been demonstrated (using synthetic micro-contractions) that the geometry also plays an important role when the degree of occlusion is fixed [4]. With the emergence of multidisciplinary areas such as biomicrofluidics, newer and more sophisticated approaches are available, where the mechanical variables associated with thrombus formation at stenosis can be studied using model experiments with controlled geometry and flow conditions. The development of micro-technologies, and in particular microfluidics has enabled unprecedented control of the experimental conditions for studying the role of hemodynamics in platelet aggregation at the micro-scale [5]. Application of these new microfluidic approaches; in combination with micro-imaging techniques applied to platelet function analysis has begun to challenge aspects of the existing models describing the early events that drive thrombus formation. Using these methods recent observations have demonstrated that modification of local hemodynamic conditions through a stenosis can trigger platelet aggregation in the absence of soluble platelet agonists signalling (ADP, thromboxane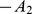 & thrombin); through the shear micro-gradient driven aggregation of discoid platelets [5]. See Fig. 1. These studies demonstrate that the accrual of discoid platelets in response to locally changing hemodynamic conditions (shear micro-gradients) is a critical driver of thrombus initiation and propagation at sites of vessel stenosis. Furthermore, these studies demonstrate that platelet-surface and platelet-platelet collisions, adhesion and associated membrane tether formation are key factors underlying discoid platelet accrual. We hypothesize that platelet tether adhesion is not simply a localized response to elevated shear and extensional forces at the stenosis, but may be a result of the cumulative effects of the entire “shear history” and concomitant cellular (particle) interactions experienced by platelets [6] and red cells as they enter the stenosis contraction, passage through the stenosis apex and exit the stenosis expansion. Key questions arising from this hypothesis are: i. What blood flow streams or regions, and as result shear-history profiles contribute to aggregate growth; and ii. How perturbations of blood flow at the stenosis can affect delivery of platelets to the adhesive substrate via mass transport and hence affect surface collision and tether formation and aggregate growth. The manner in which complex hemodynamic conditions within micro-scale stenosis affect platelet transport to thrombogenic surfaces and the effect this has on platelet activation and aggregation dynamics is poorly understood. To investigate blood cell behavior and transport under conditions of complex flow through a stenosis (patho-physiological conditions), we used a microfluidic platform that enables the discrete (tunable) control of blood flow streams over an idealized stenosis (severe micro contraction). See Fig. 2. Control of blood streams is achieved by controllably modifying the hydraulic resistance of the inlet feeders within the device, while keeping the geometrical variables of the stenosis fixed. These proof-of-concept studies have enabled us to observe an enhanced advection mechanism based on a model of competing forces: the shear gradient lift force (SGLF) and wall-effect lift force (WELF), that drive platelet transport to the stenosis apex. We further demonstrate that the balance of these forces changes as a function of platelet aggregation: During the initial stages of aggregate growth, the SGLF component predominates leading to increased cross-stream transport of platelets to the developing aggregate. Significantly, we demonstrate that platelet aggregation is self-limiting, such that once the aggregate reaches a critical size (and shape) the re-emergence of the WELF counterbalances the SGLF leading to an overall brake on platelet transport and therefore aggregate growth.

## Results

### Computational Fluid Dynamics

**Figure 3:**
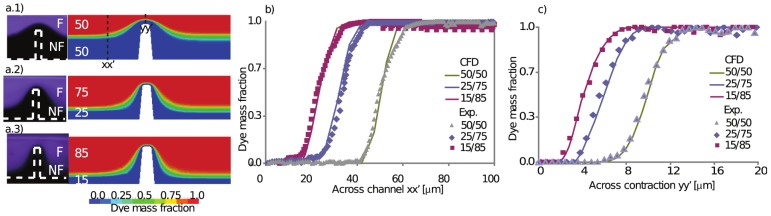
Fluidic performance using two streams of fluorescent and non-fluorescent blood, compared with CFD simulations using two species, homogeneous and Newtonian fluid, a.1) Two symmetric streams are generated of 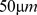. *Re = 0.78 and Pe = 770 a.2) Two streams are generated of* 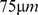 *and* 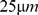 *, at the same Re and Pe number. a.3) Two streams of* 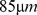 *and* 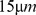 *at the same Re and Pe. b)Concentration profile across the channel from CFD and experiments with blood at* 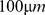 *upstream the contraction (xx). c)Concentration profile across the channel from CFD and experiments with blood at the contraction (yy).*

Fig. 3a.1) shows the flow focusing capability of the device using human blood, demonstrating that the device was able to controllably generate: two streams of laminar and equally distributed streams (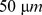/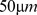) at Reynolds number of 0.78 and Peclet number of 770, two non-symmetrically focused streams (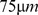/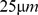 Fig. 3a.2) and, two non-symmetrically focused streams (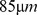/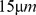 Fig. 3a.3). Comparison with CFD simulations using two species, homogeneous and Newtonian fluid demonstrates good agreement between experiments with blood and numerical calculations of streams. Figs. 3.b) presents the concentration profile across the channel from CFD and experiments at 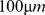 upstream contraction (xx′), and Fig. 3.c) presents the concentration profile across the channel from CFD and experiments at the contraction (yy′). It can be observed from the experiments low diffusion upstream and downstream the contraction.

### Study of platelet aggregation at micro-scale stenoses using symmetric streams

**Figure 4:**
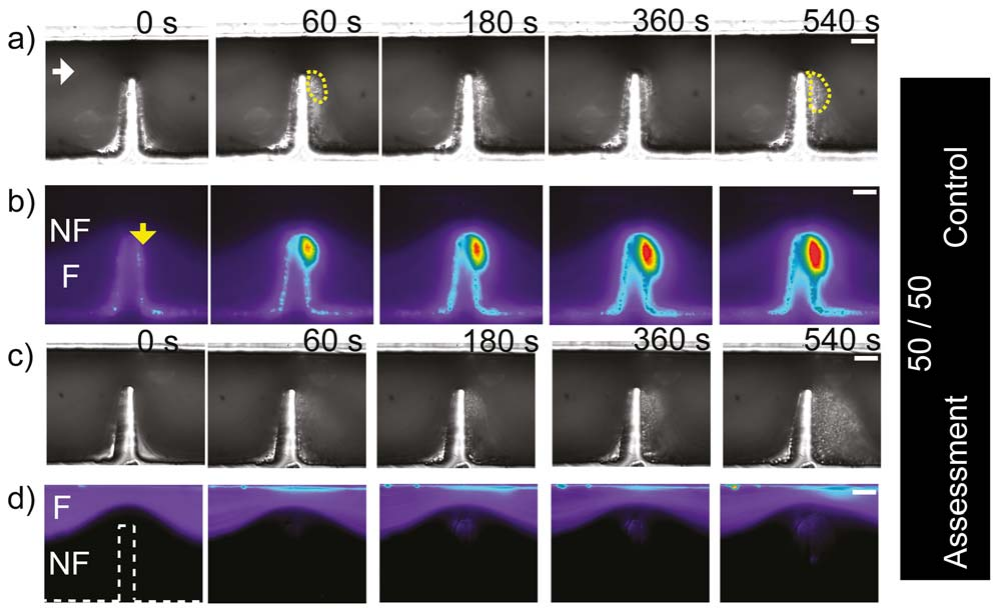
Generation of symmetric (50/50) blood streams at micro-scale stenosis. *Region 50/50, n = 3. a) DIC images of blood perfusion experiments over 10 minutes of monitoring in a device that produces two symmetrically focused streams (* 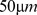 *and* 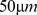 *), where the fluorescent stream is at the bottom. It can be observed that an aggregate formed downstream of the contraction using two streams. b) Representative (n = 3) epi-fluorescence image of the same experiment as (a)), a strong fluorescent platelet incorporation into the developing aggregate was observed which corresponds to the DIC visible aggregate. c) Same experiment as a) but the fluorescent stream was located at the bottom. d) Representative fluorescence image of the same experiment as (c)), showing that no fluorescently label aggregation occurred downstream of the contraction using two stream. (White bar: Scale bar* 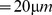 *.)*

In order to simplify our proof-of-concept studies and to isolate the mechanical effects of blood flow from biochemically driven platelet activation, all experiments were performed in the presence of pharmacological inhibitors of the canonical platelet amplification loops as was described in the methods section. For the first experiment with blood, a two flow geometry as presented in Fig. 2 was used to generate two 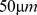 symmetrically focused streams upstream of the stenosis leading to two symmetric 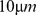 streams at the apex of a severe micro-contraction. Figs. 4a)–d) show DIC (Differential Interference Contrast microscopy) and epi-fluorescence images of whole blood perfusion experiments over 10 minutes. Figs. 4a)–b) presents a control experiment performed to corroborate platelet aggregation response is not affected by splitting the blood sample between two streams. Whole blood was perfused in both streams and the bottom stream was in addition premixed with a fluorescent dye (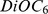 (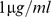) 10 mins rest) to label the platelets. Interrogation by DIC imaging demonstrated robust platelet aggregation within the downstream expansion zone of the micro-contraction. Fig. 4b) shows epifluoresence imaging demonstrating homogeneous distribution of 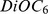 labelled whole blood within the bottom 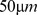 of the micro-channel which narrowed to 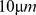 at the apex of the stenosis. Strong fluorescent platelet incorporation into the developing aggregate was observed which corresponds to the DIC visible aggregate Figs. 4a)–b) and is equivalent to our previously published results [4]. In reciprocal experiments where 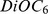 labelled blood was confined to the top 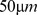 stream, significantly, no fluorescence incorporation into the developing platelet aggregate was observed for this case (Fig. 4d). This data suggests: i) that only platelets within streamlines biased to the micro-contraction geometry side of the micro-channel took part in platelet aggregate formation, or equivalently the platelets from upper regions did not take part in the aggregate, ii) from Figs. 4c)–d) it is clear that the two streams remained distinct and un-mixed both upstream and downstream of the contraction, indicating that for these stream widths, the effect of cell-cell interaction/collisions is insignificant even though flow perturbations due to such collisions could be expected due to the fact the micro-contraction is on the same order of magnitude of an undeformed red cell (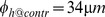, 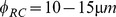 respectively) which are present in both streams.

### Study of platelet aggregation at micro-scale stenoses using asymmetric streams

**Figure 5:**
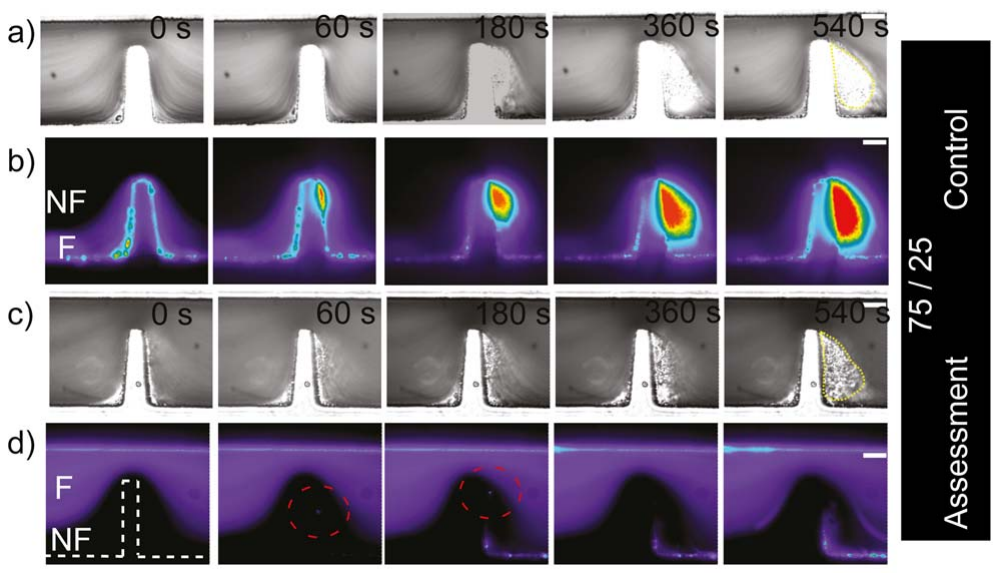
Generation of asymmetric (75/25) blood streams at micro-scale stenosis. *Region 75/25, n = 3. a) Representative (n = 3) DIC images of blood perfusion experiments over 10 minutes of monitoring on a device that produces two non-symmetrically focused streams (* 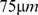 *and* 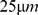 *). It can be observed that an aggregate formed downstream of the contraction using two streams. The yellow polygon indicates platelet aggregates. b) Representative (n = 3) epi-fluorescence images of the same experiment as (a)), showing that an aggregate was formed downstream of the contraction using two streams. The white arrow indicates flow direction. c) DIC images of the same experiment as a) but the fluorescent stream was located at the top wall. d) Representative (n = 3) epifluorescence images of the same experiment as (c)), showing that an aggregate was formed downstream of the contraction. (White bar: Scale bar* 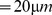 *.)*

**Figure 6:**
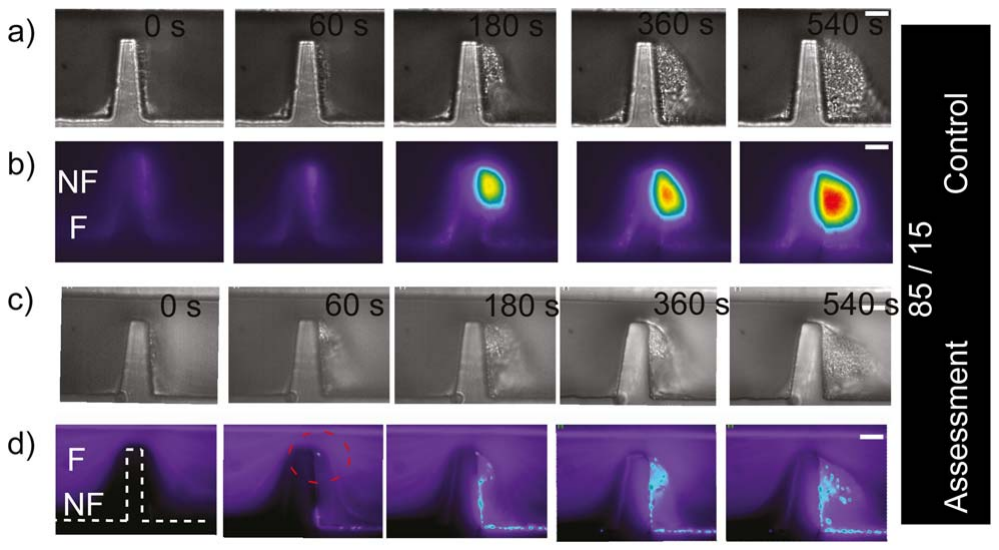
Generation of asymmetric (85/15) blood streams at micro-scale stenosis. *Region 85/15, n = 3. a) Representative DIC images of blood perfusion experiments over 10 minutes of monitoring in a device that produces two non-symmetrically focused streams (* 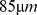 *and* 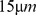 *). It can be observed that an aggregate formed downstream of the contraction using two streams. b) Representative (n = 3) epi-fluorescence images of the same experiment as (a)), showing that an aggregate was formed downstream of the contraction using two streams. c) DIC images of the same experiment as a) but the fluorescent stream was located at the top wall. d) Epi-fluorescence imaging of the same experiment as (c)), showing that an aggregate was formed downstream of the contraction. Note, contrary to previous experiments, fluorescent platelet incorporation started to be evident at initial stages of platelet aggregation (60 s, 180 s). (White bar: Scale bar* 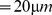 *.)*

**Figure 7:**
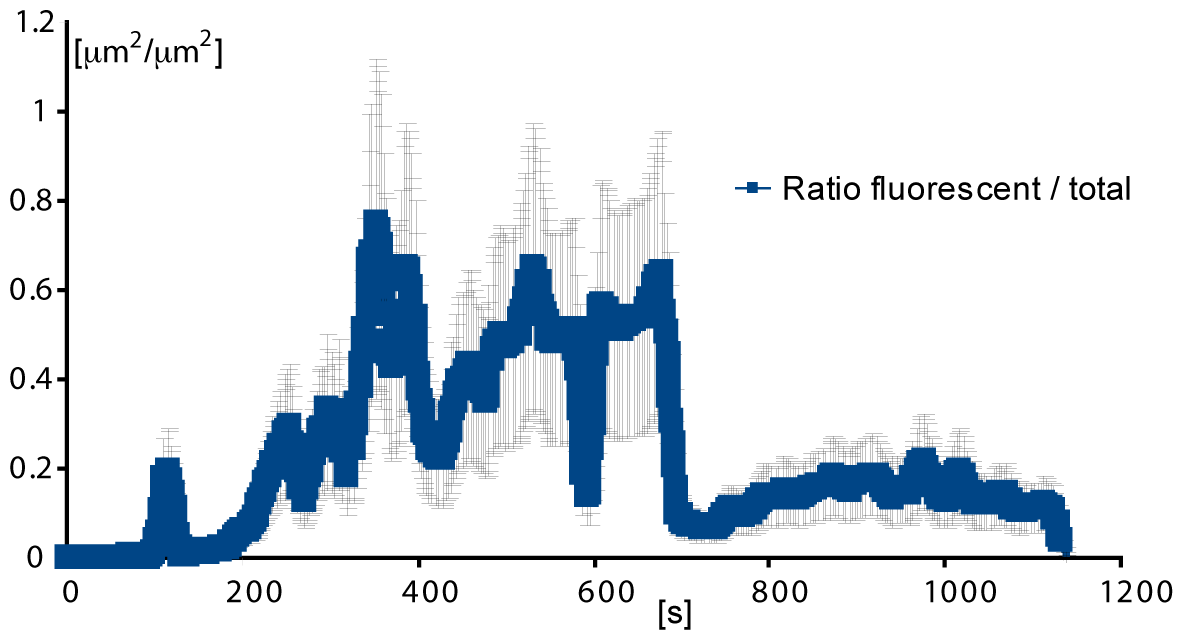
Measurement of aggregation traces over time. *Left: Aggregate size over time. The orange trace represents the total surface area of the aggregate measured from DIC imaging. The blue trace (behind the error bars in black) represent the total surface area of the aggregate composed by fluorescent platelets. n = 3. Right: Ratio of the fluorescent surface area component over the DIC visible surface area, presented in* *Fig. 5d* *, Showing that the phenomenon of crossing streams of fluorescent platelets coming from the upper layer to the non-fluorescent bottom stream, initiated only once the developing aggregate had reached a critical surface area (left100 s), and that this phenomenon was dynamic with time. As a particular example, the size of platelet aggregate which had incorporated fluorescent platelets was comparable with the total size of aggregation at 300 and 500 seconds, but was less significant at later stages such as at 1200 seconds. 5.*

The experiments presented in the previous section were useful as a proof of concept to visualize different blood streams, to begin gaining insight into which components of the blood flow contribute to the aggregate as also to observe cell interaction. To further resolve the platelet streams contributing to aggregation and explore in greater detail the cellular interactions occurring within defined stenosis, we conducted a series of experiments to generate asymmetric streams in a ratio of 75∶25 and then 85∶15. Figs. 5a)–b) show a control experiment (equivalent to Fig. 4a)–b)) to test whether the platelet aggregation response was affected by the technique of splitting the blood sample between streams, where labelled blood was confined to the bottom 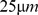 stream and unlabelled blood was confined to the top 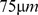 stream. It can be observed from the DIC images that aggregation was effectively formed within this device. Epi-fluorescent images show strong fluorescent platelet incorporation into the developing aggregate. This control experiment clearly showed that platelet aggregation can be achieved within this device. Fig. 5c)–d) correspond to the reciprocal experiments to those of Figs. 5a)–b), with fluorescently labelled blood in the top 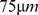 stream and unlabelled blood in the bottom 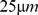 stream. Almost no fluorescent platelets are evident within the aggregate, demonstrating that platelets within the top 75% of streamlines made no significant contribution to aggregate formation. Similarly to the previous symmetric experiment this data suggests, i) that only platelets within streamlines biased to the micro-contraction geometry side of the micro-channel, but now in a reduced region of 25% took part in platelet aggregate formation (compared with our last experiment (of 50%)), ii) in terms of cell-cell interaction, it was interesting to observe that perhaps a single fluorescent platelet was able to take part on the non-fluorescent aggregate (red dotted circle at Fig. 5d-60 and 180 s). This could suggest that the thickness of the streams (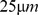) is starting to become relevant to observe cell-cell interaction/collisions allowing platelets to cross streams near the contraction. It should be noted also, that after 3 minutes of perfusion, some level of fluorescent platelet adhesion was observed, with platelets forming a thin mono-layer immediately downstream of the micro-contraction geometry. This low level incorporation may have been due to the generation of a flow recirculation region adjacent to the platelet aggregate that should develop as a function of aggregate size. Some degree of recirculation and/or disturbed flow was evident in the DIC imaging experiments (see section 2.6). In order to further resolve the contribution of platelet streamlines to aggregate development we conducted perfusion experiments to generate asymmetric streams in a ratio of 85∶15 at the stenosis. At the apex of the micro-contraction, this equates to a lower focused stream width of 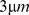 (equivalent to approximately one platelet diameter, and 1/5 of a red cell diameter). Figs. 6a)–b) show a control experiment (equivalent to Fig. 4a)–b)) to test whether platelet aggregation response was affected by splitting the blood sample between these two channels. As before, platelet aggregation is clearly evident. It can be observed also, that a significant proportion of the total aggregate mass developed through the accrual of platelets that were effectively focused within 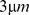 of the micro-geometry wall; at the apex of the stenosis. Reciprocal experiments were performed where the upper 85% of blood was fluorescently labelled. These results are presented in Fig. 6c)–d). Interestingly, these demonstrate that fluorescent platelets appeared within the aggregate which much more frequency than for wider flows (compare Fig. 6d) 60–180 s to Fig. 5d) 60–180 s), which suggest the platelets from the upper stream were able to cross streams more easily with such a thin lower stream (Fig. 6d 60 s to 540 s). Similarly to previous experiments this data now suggests: i) that platelets within streamlines biased to the micro-contraction geometry side of the micro-channel, from a region of 15% were not the only platelets taking part in aggregate formation (there was also a significant fluorescent component);, ii) we could say terms of cell interaction, we effectively observed that there was a mechanism enabling platelets to cross streams in a consistent manner from the fluorescent region to the non-fluorescent region (red dotted circle at Fig. 6d-60 s, 180 s). Fig. 7 shows the ratio of the fluorescent surface area component over the DIC visible surface area, and enables quantitative distinction of the proportion of total aggregate size (DIC visible) and the fluorescent aggregate surface area component for the last experiment presented (Fig. 6c–d). It can be observed that the phenomenon of crossing streams of fluorescent platelets coming from the upper layer to the non-fluorescent bottom stream, initiated only once the developing aggregate had reached a critical surface area (Fig. 7.300 s). Furthermore, this data demonstrates that platelet incorporation through streamline crossing was dynamic with time. As a example, the size of platelet aggregate which had incorporated fluorescent platelets was comparable with the total size of aggregation at 300 and 500 seconds, but was less significant at later stages such as at 1200 seconds. Taken together this data clearly illustrates a nonlinear process where aggregate development perturbs local blood flow streamlines leading to enhanced advection of platelets to the growth face of the aggregate, while further growth and stabilization of the thrombus reduces the perturbation of the flow and hence there is reduced platelet advection.

### Role of the hematocrit on platelet aggregation response

**Figure 8:**
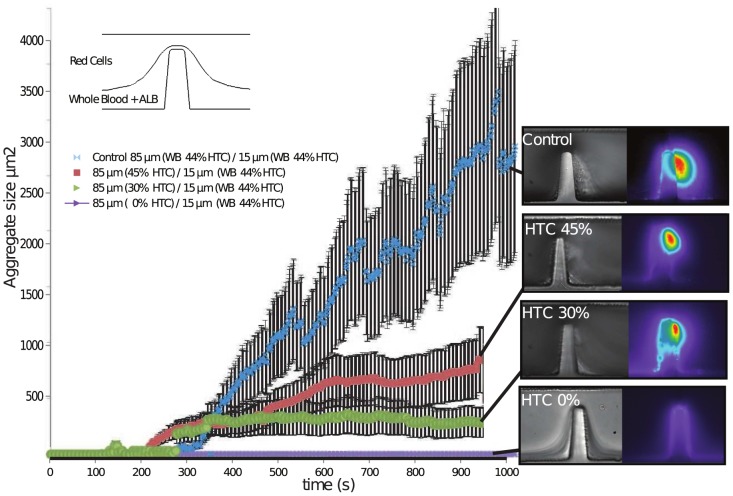
Generation of asymmetric (85/15) blood streams at micro-scale stenosis to investigate role of the upper blood stream as a function of (hematocrit) on platelet aggregation. *Platelet aggegation response was found to be a function of the hematocrit in the upper layer. The hematocrit in the upper stream of 85% (* 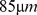 *) was modified from 0% (buffer solution) to 45%. The bottom stream of 15% (* 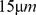 *) was infused with whole blood with the platelet inhibitors of aggregation (Amplification loop blocked ALB). The platelet aggregation response is effectively modulated by the upper layer and its concentration of red cells.*

In order to investigate the role of the hematocrit in the upper streams and the effect of red cell interactions at the stenosis, model experiments on the microfluidics platform were conducted where the upper layer was changed as a function of the amount of red cells suspended in solution. The hematocrit in the upper stream of 80% (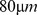) was modified from 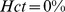 (buffer solution) to 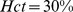 and 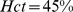. The bottom stream of 20% (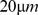) was infused with whole blood with the platelet inhibitors of aggregation. Figure 8 shows the aggregation traces of these analysis and a segment of the imaging of the experiment at the last stages (10 min), it can be observed that the platelet aggregation response is effectively modulated by the upper layer and its concentration of red cells, where at 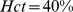 of hematocrit no aggregation was found. At 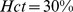 some platelet aggregation can be observed and at 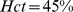 almost a two fold up in size. However it should be noticed that even at 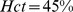 the size of the platelet aggregate was not as big as the one found in the control experiments where in both streams whole blood was perfused. This suggests that not only the red cells, but platelet from upper layers, and proteins, may play a role in aggregate formation.

### Role of early aggregate development on enhanced platelet advection

**Figure 9:**
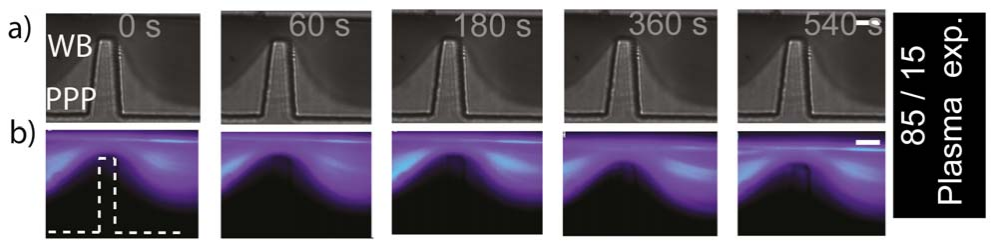
Generation of asymmetric (85/15) blood streams at micro-scale stenosis to investigate the role of early aggregate development. *Top stream (85): labelled whole blood. Bottom stream (15): Autologus platelet-poor-plasma (PPP). a) Representative DIC images of blood perfusion experiments over 10 minutes of monitoring in a device that produces two non-symmetrically focused streams (* 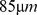 *and* 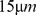 *). It can be observed that contrary to previous experiments, no aggregate was formed downstream of the contraction. b) Representative (n = 3) epi-fluorescence images of the same experiment as (a)). No aggregate was formed downstream of the contraction. This data demonstrates that initial aggregate formation was driven by platelets skimming within* 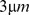 *of the stenosis apex.*

To explore the effect of platelet stream crossing and to investigate the role of early aggregate development, we conducted a series of asymmetric streams experiments in which the upper 85% of flow was 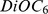 labelled whole blood and the lower 15% was comprised of autologus platelet-poor-plasma (PPP). Figure 9a–b demonstrates that in the absence of platelets within 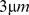 of the stenosis apex no fluorescent platelet incorporation was observable following 10 min of perfusion. This data directly demonstrates that initial aggregate formation was driven by platelets skimming within 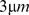 of the stenosis apex. Furthermore this data shows that in the absence of initial aggregate development no advection of platelets to the adhesive channel wall occurred suggesting that platelet advection is triggered once the developing aggregate reaches a critical size.

### Vortex formation as a function of platelet aggregate size

**Figure 10:**
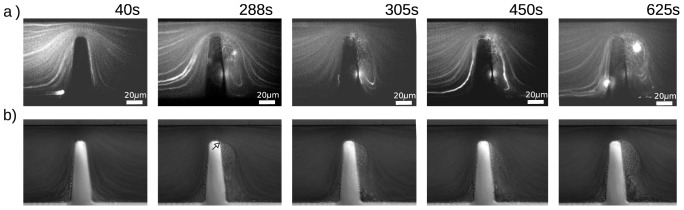
Investigation of vortex formation on the microcontraction as a function of aggregate growth at 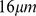 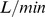 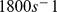, Reynolds 0.78.a) Epi-fluorescent images show the path of the tracers (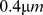 microparticles). *b) DIC images of the experiment. Notice that the platelet aggregate starts growing immediately after the expansion, downstream of the tip of the contraction, arrow in b) at 288 secs). See (Video S1).*

The experiments presented in Section 2.3 showed the formation of a platelet aggregate as a function of the shear microgradient at the deceleration zone of the contraction, it was also noted that some vortex formation was observed at the corner downstream once the aggregate reaches a sufficient size. Vortex formation, even at Reynolds numbers approaching zero, has been reported in several investigations [7], [8]. To investigate vortex formation induced by the aggregate in our investigation, we performed a series of experiments to visualize flow streamlines traced by 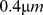 diameter fluorescent microparticles of (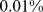) dispersed in whole blood. The flow conditions were kept the same of those used in Section 2.3 (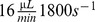, Reynolds number 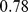), using a single flow geometry. Simultaneous acquisition on two channels (FITC and DIC) was performed during the experiment, to visualize the streamlines of the fluorescent particles and the development of the platelet aggregate respectively. Figure 10 presents the results of the experiments. Figure 10 a) presents epi-fluorescence images showing the path of the tracers (microparticles). Figure 10 b) presents DIC images of the aggregate formation for comparison. It can be observed that early during aggregation [Fig. 10a), at 40 s], the streamlines are parallel and no vortex is evident. As the aggregate grows [as clear in the DIC image of Fig. 10 b), at 288 s], there is a vortex region at the corner downstream, at the bottom of the aggregate [as evidenced by the bright recirculating trace in Fig. 10 a) at 288 s and also evident at 305, 450 and 625 s]. From the DIC images (Fig. 10b), it can be observed that there is strong platelet accumulation at the early stages of the experiment (288 s), and an accumulation of larger red cells under the aggregate as a consequence of the vortex region induced by the aggregate. Notice that the platelet aggregate starts growing immediately after the expansion (at the tip of the contraction, see arrow, Fig. 10b 228 s) where no vortex formation was observed.

## Discussion

**Figure 11:**
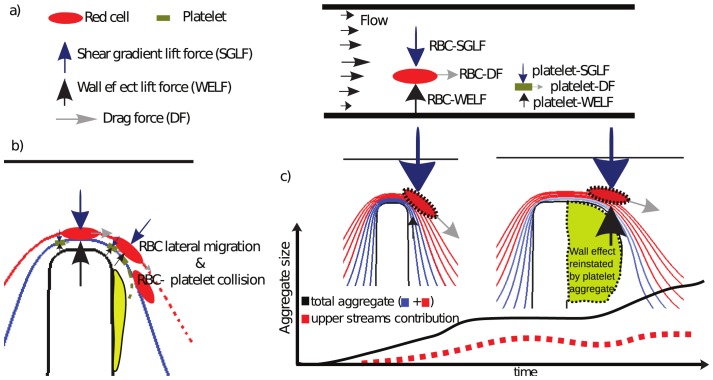
Proposed mechanism of platelet transport to the wall induced by red cell lateral migration. *a) Forces experienced by blood cells: drag and lift forces. The shear gradient lift force (SGLF) directs cells towards the wall. The wall-effect lift force (WELF) directs cells away from the wall. b) Red cell at the expansion may not follow their original trajectories, migrating from their original streamlines which produce an intersection with platelet trajectories sending them to the wall. This is suggested by a calculation of the Stokes number (mismatching trajectory effects), which is on the order of 190 times higher for red cells. This migration to the wall from the red cell is a consequence of the interaction of lift forces at the stenosis. As cells approach the expansion the wall-effect lift diminishes and the shear-gradient lift induces cells to migrate laterally across streamlines towards the wall, red cells migrate to the wall, colliding with platelets, increasing platelet transport to the wall, platelet contact and therefore aggregation. This is supported by the experiments presented in* *Fig. 6d* *) where fluorescent platelets migrated to the non-fluorescent stream. c) Representation of the role of the aggregate in re-establishing the balance of lift forces over a typical aggregation trace curve (as a function of area over time) of an experiment similar to* *Fig. 7* *. We hypothesize that at later stages of platelet aggregate growth, the size of the platelet aggregate enables it to reinstate the wall-lift effect, regenerating the balance between wall-lift force and shear-gradient lift force, minimizing red cell to platelet interactions/collisions and suppressing advective transport of platelets to the aggregate surface, as is supported by experiments, see* *Fig. 6d* *) and* *Fig. 7* *right (800 s).*

The experiments presented in this manuscript demonstrate the utility of microfluidics in resolving the blood streams that effectively contribute to platelet aggregation at a defined stenosis. Significantly, by progressively biasing and resolving the fluid streams we demonstrate that only 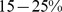 of the blood stream proximal to the stenosis geometry contributes to aggregate development (where study reports indicate platelet density at its highest [9]–[13]). This equates to a fluid layer of 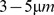 at the apex of the contraction; approximating the length scale of discoid platelets (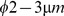) and well below the size of erythrocytes (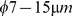). Experiments using a combination of fluorescently labelled blood where streams were asymmetrically focused to an inflow width of 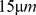 (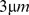 at the apex) suggest (by extrapolation) that platelets undergo dynamic changes in advective transport within a 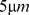 fluid layer that is dependent on pre-existing aggregate formation. Accrual of fluorescently labelled platelets at the outer surface of developing aggregates was only observed beyond 3 minutes of perfusion, suggesting that platelet aggregates must reach a threshold size and shape before platelets from a cross stream can be captured via enhanced advection to the aggregate within this relatively narrow 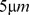 fluid layer. This experiment suggests that as the stream thickness is reduced upon entry into the stenosis, the size of blood cells and pre-existing aggregates becomes increasingly important, such that cross-stream platelet-platelet interactions or collisions modify platelet trajectories leading to enhanced advection of platelets to the wall and increased collisions with the aggregate surface. Based on the experimental observations presented above, we have effectively defined a significantly narrow stream of blood that contributes to aggregate growth. A key question arising from this study is how the shear stress history of platelets within a constrained stream affects aggregate growth. Applying Computational Fluid Dynamics calculations of strain-rates, we can estimate that the minimum stress history range that produces aggregation at the stenosis in our device was the streamline within 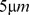 of the stenosis apex. Platelets within this stream experience shear rates on the order of magnitude similar to 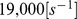 at stenosis with a decrease of 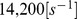 and 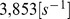 at 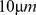 and 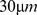 downstream of the apex, respectively. Further experiments are needed to refine the minimum shear threshold of aggregation using wider gaps at the contraction. Based on these observations and our previously published studies, we hypothesize that these rapidly varying shear rates at the stenosis results in high deformation rates that promote the extension of filamentous membrane tethers from discoid (resting) platelets, increasing the probability that platelet-surface and platelet-platelet collisions will result in semi-stable platelet capture, while the rapid decrease in shear stress and velocity, post apex, promotes stabilized aggregation. Significantly, we hypothesize that collisions with the red cell fraction of blood is a key mechanism by which discoid platelets are transported into these high shear conditions and subsequently decelerated in close proximity to the wall, increasing the frequency of surface contact and therefore tether based adhesion formation. This hypothesis could explain the platelet behaviour observed from the apparent streamline crossing (Fig. 6d-60 s, 180 s), as it will be explained below. Blood cells in flow experience both, shear and normal stresses acting over their surfaces producing drag and lift forces (parallel and perpendicular to the main flow direction, respectively) [14]–[17]. In a channel, the drag force (Stokes), causes blood cells to travel in the flow direction, at the average fluid velocity, while lift forces (wall repulsion and shear gradient forces) cause blood cells to move laterally (migrate) with wall-repulsion lift force pushing cells away from the walls, and shear-gradient lift forces pushing the blood cells away from the centre of the channel. As a function of this, blood cells will migrate to an equilibrium position within fluid streamlines, where the lift forces are balanced [18], [19]. These forces originate mainly from gradients of velocity acting on the cell surface, however, particle and fluid properties also play an important role in the magnitude and direction of these forces [20]–[22]. In the case of blood flow, it is known that as a result of these forces, red blood cells tend to migrate towards the centre and platelets migrate towards the wall [9]–[13], [23], [24]. Because of their size, higher deformability and higher concentration under normal physiological conditions, the hydrodynamic forces acting on red cells are in general much larger than the forces acting on platelets, which are smaller and more rigid [25]. To explain the behaviour of the platelet response in Fig. 6d-60 s, 180 s) and Fig. 8, we can visualize the forces that the blood cells experience. Fig. 11 a) presents a free body diagram of these forces. The shear-gradient lift force (SGLF) directs cells towards the wall (pushing them away from low shear gradients) while the wall-effect lift force (WELF) directs cells away from the wall (pushing them away from high shear gradient at the wall). These forces are present in the contraction where they interact (it is uncertain if they are at equilibrium), but as cells approach the stenosis expansion these forces change radically and the wall-effect lift (WELF) diminishes and the shear-gradient lift force (SGLF) pushes the cells towards the wall. The magnitude of this SGLF force is related to the particle diameter 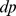 and the channel width W. When 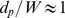, the SGLF scales as 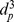, when 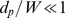, the SGLF scales to 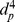 [26]–[28]. This means small particles, such as platelets (or fluid tracers such as microparticles used in image velocimetry techniques), in the expansion may be able to maintain their streamlines because they experience minimal shear-gradient lift force, but this may not be true for erythrocytes, which are an order of magnitude larger. The related lift force imbalance in the expansion may be much larger for erythrocytes than platelets (we estimate two or three orders of magnitude), which in addition experience higher levels of deformation than platelets. Furthermore, even though a simplistic estimation of the particle Reynolds number (inertial/viscous effects) and Stokes number (trajectory mismatch effects) at the contraction suggests that it should be possible for erythrocytes to adapt instantly to the fluid trajectory (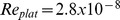, 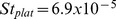, 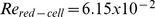, 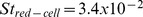, the ratio between the Stokes number for erythrocytes and platelets (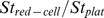) is on the order of 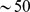, which means any possible mismatching to the fluid trajectory should take place firstly for erythrocytes (assuming erythrocytes diameter 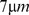, discoid platelet diameter 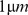, velocity at the contraction of red cells 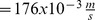, platelets 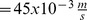). We hypothesize that this imbalanced lift force effect at the expansion produces minimal changes in platelet trajectories but far more significant effects on erythrocytes trajectories, inducing eventual collisions between erythrocytes and highly strained platelets, which are sent to the streamlines close to the wall. Micron-scale transport of platelets from higher to lower velocity streamlines within a short distance of the aggregate surface not only causes platelets to experience an increase in deformation rates but should also lead to an overall reduction in velocity, effectively increasing residence times at the aggregate surface, increasing the probability for tether formation and surface capture (see Fig. 11b)). Our mixed blood and plasma experiments (Fig. 9a)–b)), demonstrated that depletion of platelets within streamlines at the channel surface (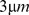 from the stenosis apex) completely inhibits all aggregate growth despite the fact that the normal ensemble of blood plasma proteins (von Willebrand€s factor and fibrinogen) are present in the plasma layer. These experiments reinforce the observation that advective transport of platelets from streamlines outside of this fluid layer is critically dependent on the formation of an initial platelet aggregate. A key finding to arise from our experiments is that the observable increase in platelet advection to the surface of developing aggregates is transient in effect. Fig. 7 demonstrates that under the defined shear micro gradient conditions utilized in this study, cross stream platelet capture within 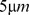 of the aggregate surface diminishes at approximately 4–6 minutes of blood perfusion. This observation suggests that as aggregate size and shape progresses, the initial hemodynamic conditions that led to enhanced advection and stream crossing within the fluid layer diminish; with ongoing aggregate growth due to capture of platelets skimming within 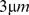 of aggregate surface. We hypothesize that at later stages of platelet growth, the size and equilibrium shape of the platelet aggregate enables it to reinstate the wall-lift effect, re-establishing the balance between wall-lift force and shear-gradient lift force. At this state of aggregate growth, erythrocytes and fluorescently labelled platelets return to unperturbed parallel streamlines (Fig. 11c)). This aggregate dependent restitution of WELF effectively minimizes erythrocytes to platelet interactions/collisions and suppresses advective transport of platelets to the aggregate surface. This important role of the WELF also explains the role of the hematocrit in the aggregate size, as was demonstrated on Fig. 8 by creating a smashing effect from the red cells on the upper streams to the bottom streams of platelets. This smashing effect enhances the transport of platelets to the wall. While these experiments strongly suggest that variation in streamline hemodynamics and platelet advection may be key determinants of aggregate growth, biochemical changes and modification of platelets already incorporated at the aggregate surface cannot be ruled out as contributing factors. A number of studies suggests that both platelet activation status and exposure to fluid shear can lead to surface modification and shedding of platelet surface expressed adhesion proteins that may contribute to the finite aggregate growth observed in our experiments [29], [30]. Furthermore, shear stress is a known modulator of von Willebrand factor (vWF) structure, with steady-state elevation of shear stress demonstrated to vWF multimers exposing platelet binding domains. The way in which shear micro-gradients affect vWF conformational structure is an unexplored area that may have a significant impact on the feedback effects of aggregate size and shape on platelet recruitment and aggregation. Future studies will address the effects of the shear micro-gradient environment on both aggregate surface modification (receptor shedding) and vWF conformation and delivery to the growth face of developing aggregates. The development of recirculation zones as a function of blood flow through stenosis has been hypothesized to be significant contributor to thrombus growth, through the entrapment of blood cells and localized concentration of secreted biochemical agonists such as ADP. In our experimental system initial aggregate formation is observed to occur in the absence of flow recirculation (vortex). As platelet aggregation proceeds and once the aggregate reaches a sufficient size (Fig. 10a,b 288), vortex formation is observable with a clear zone of recirculation at the bottom of the forming aggregate. We speculate that this interaction may enhance the transport of single platelets to the bottom of the already initiated thrombus and may also effectively deliver aid blood borne chemicals agonists to this region of platelet aggregation, as has been suggested by many others [31]–[40]. Vortex formation may constitutes a secondary process that may serve to accelerate platelet aggregation once the initial platelet mass has been established, however the initiation of the aggregation is due to the shear gradient mechanism. This secondary phenomena may be responsible for the accelerated aggregation observed in the aggregate trace plot of Fig. 7, where once a reasonably stable size is reached (for example at 360 s) aggregate growth appears to accelerate.

## Conclusions

This paper presents model experiments on a microfluidic platform incorporating hydrodynamic flow focusing to examine blood cell transport investigating the mechanical flow processes governing pathological platelet aggregation at stenosis (micro-contractions). These initial proof-of-concept experiments suggests that aggregate growth in acute stenosis can generate enhanced advective transport zones within the local flow effectively increasing platelet mass transport to the wall, further accelerating aggregate growth. This feedback effect of platelet aggregation on advective transport may be a function of the change in angle of deceleration of the stenosis or the already formed aggregate. Future studies will focus on further delineating this phenomenon and in particular will focus on the effect of initial streams acceleration as a significant parameter determining platelet aggregation dynamics. We have demonstrated the importance of the effects of hemodynamic forces present in a stenosis driving platelet aggregation. The experiments presented in this manuscript reveal a significant role of role of lift forces at stenosis. These forces appear to be important in modulating the delivery rate of platelets to the vessel wall. Delivery rate is mediated by the action of red cells which because of their relative size experience a higher imbalance of lift forces in comparison to platelets. We hypothesize that this imbalance of forces, is a generalised phenomena and consequence of stenosis and that the experimental approach outlined in this study will lead to a greater understanding of the mechanisms underlying shear-gradient dependent discoid platelet aggregation. These new experimental insights suggest a complex time varying role of enhanced advection through particulate flow, in controlling the extent and rate of platelet aggregation. Although it may be speculative to apply the insight gained here to more inertial dominated flows, a more detailed understanding of these aggregation phenomena should greatly extend our understanding of the mechanical flow processes governing pathological thrombus formation in the context of coronary artery disease and stroke, with particular relevance to inertially dominated stent related thrombosis.

## Materials and Methods

### Device design, fabrication and operation

Fig. 2 presents the overall device design and layout. The microfluidic device was designed to minimize contact with glass or plastic materials and long dead volumes (all of which can lead to pre-activation of blood cells and modification of blood samples) by using a reservoir where the blood sample is loaded close to the micro-contraction (on-chip sampling). As the micro-channels are designed to mimic haemodynamics in-vivo, the dimension of the channel in its straight section was 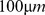
×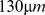. As a starting point to develop our microfluidic platform we focused on using a previously validated shear micro-gradient geometry representing severe stenosis that has been demonstrated to produce stable platelet aggregation (stenosis of 80%). A micro contraction with an idealized geometry (85 degree) to maximize platelet response was microfabricated to mimick the hemodynamics conditions of the induced stenosis in the in-vivo experiment.[4], [5]. It was demonstrated on [4] that a gentle slope (60 degree-angled geometry) was also able to produce platelet aggregation in a similar amount than the geometry presented here. However it was also shown that the 90 degree geometry produced a more stabilized platelet aggregate. The flow rate was set to 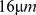
L/min to produce strain-rates of 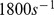 upstream the micro-contraction to mimic the strain-rates of small arteries/arterioles[4], [41]. Blood flow was induced using negative pressure (suction) with a single syringe connected to the outlet port. Different widths of the focused stream () was achieved by changing the hydraulic resistance of the inlet feeder channels as presented in the (Text S1). A manually operated pneumatic valve was incorporated into the chip using multi-layer soft lithography [42] to allow easy purging of the fluid lines as air bubbles can lead to resistance changes and disruption of streamline control. Blood perfusion studies through the device were carried out at the Australian Centre for Blood Diseases, Monash University, using hirudin (800 U/ml) anti-coagulated whole blood, taken from consenting human donors. Approval for these studies was obtained from the Monash University Standing Committee on Ethics in Research Involving Humans. All donors signed a consent form where they are made aware of the research project. To document these process we kept signed copies of all donor consent. The Monash University Standing Committee on Ethics in Research Involving Humans approved this procedure. Whole blood samples were incubated at for 10 minutes with the lipophylic membrane dye 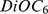 (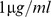) [Molecular Probes]. Flow in the device was induced by a Harvard 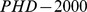 syringe driver connected to the outlet channel in the PDMS block using a Becton Dickinson 3 ml glass syringe attached to 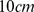 of Tygon (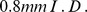) tubing to minimize pulsatility. Blood samples were introduced into the micro-channels via the 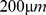
L reservoir cut into the PDMS block at the channel inlet. Platelet aggregation was monitored via epi-fluorescence (Sutter DG4 Xenon arc lamp [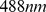] light source) using an 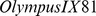 inverted microscope with a 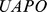 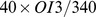 objective and attached Hamamatsu Orca ER CCD with an exposure time of 30 ms. Image acquisition was controlled through Metamorph 6.0 (multi-dimension acquisition). Blood flow was observed within a focal plane approximately 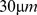 above the cover-slip wall of the channels. Fluorescently labelled platelet aggregates were segmented via intensity thresholding and the threshold area determined on a frame by frame basis in Metamorph 6.0. In order to simplify our proof-of-concept studies and to isolate the mechanical effects of blood flow from biochemically driven platelet activation, all experiments were performed in the presence of pharmacological inhibitors of the canonical platelet amplification loops: (MRS (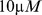) to block 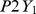 signalling, 2MeSAMP (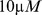) to block 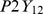 activation and Indomethacin (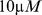) to block 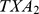 generation.

### Computational Fluid Dynamics

In order to model the flow focusing behavior of the micro-fluidic devices, the mass transport equation for two species was solved numerically. For the case of this paper, no chemical reaction was considered and the only phenomena present for the chemical species was the transport of mass and momentum. The governing equation of the mass transport for different species can be expressed as:
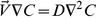
(1)Where 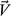 represents the velocity vector, C represents the concentration of species, D represents the diffusion constant. The velocity field and its derivatives were calculated solving the mass conservation equation using FLUENT 6.0 (Fluent USA, Lebanon, NH) based on a finite volume scheme. A 3D model was used with a double precision segregated algorithm. The flow was modelled as laminar, steady and incompressible. Two chemical species were used as fluids, with a density of 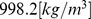, a viscosity of 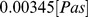 and a diffusion constant was assumed as 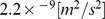 [4], [43], [44]. The geometries were discretized with hexahedral elements using ICEM (Ansys). A mesh independence analysis was performed using a two dimensional model to investigate the mesh density needed across the channel [8]. In order to analyze the relations and the relative importance of the phenomena affecting the microfluidic devices presented, several dimensionless parameters were considered. The Reynolds number (Re) defined as the ratio of inertial forces to viscous forces, (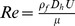) where 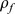 is the density of the fluid, 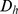 is the hydraulic diameter of the channel (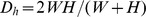), 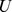 the average velocity of the fluid in the channel and 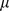 is the dynamic viscosity. The particle Reynolds number (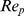) measures the influence of the inertial and viscous forces from the fluid on the particle, (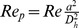). In order to quantify the transport phenomena, the Peclet number which measures the advection effects relative to the diffusion (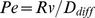), where 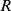 is the radius of the channel or blood vessel, 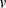 is the average velocity, and 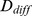 is the diffusion coefficient. Finally for a particle in an accelerating flow, the Stokes number measures how quickly the particle adjusts to changes in the flow, which is useful to study trajectory mismatch between particles and fluid. This is defined as the ratio between the particle relaxation time 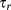 and to the characteristic time of the flow 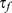 [45], (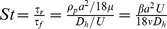) where 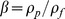 and 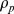 is the particle density.

## Supporting Information

Text S1 Vortex formation as a function of platelet aggregate size.
(DOCX)

Video S1 Supplementary Material.
(AVI)
