# Supplementary material for: An Investigation on Platelet Transport during Thrombus Formation at Micro-Scale Stenosis
Source: PLoS One. 2013 Oct 23;8(10):e74123. doi: 10.1371/journal.pone.0074123 (PMC3806794; doi:10.1371/journal.pone.0074123)
Supplement: Text S1 — Vortex formation as a function of platelet aggregate size. (DOCX) [file pone.0074123.s001.docx]

## Supplementary Material

### Fabrication

The devices were fabricated from PDMS (Sylgard) using a two-step molding process. Each mold was defined photo-lithographically. The first mold contained the flow layer, including the micro-contraction, and the second mold the control layer and reservoirs. The micro-contraction defined in first mold was produced using a high resolution chrome mask (250*nm*) on soda lime glass to achieve well defined features and straight side walls of SU-8 3050 (Microchem, Newton, MA) photoresist, (as is described in [4]) and was spin coated with PDMS using a spread cycle of 300 rpm and 100 rpm/s for 10 seconds and a second cycle of 550 rpm and 300 rpm/s for 30 seconds, in order to achieve a PDMS film of 250 μ*m* thickness with good uniformity. The second mold was fabricated using a low resolution mask (2400*dpi* ~30μ*m*) printed on a film and using a similar photo-resist thickness to the first mold. A Perspex (PMMA) enclosure 6 mm of thick was attached to the second mold to allow formation of a 6*mm* thick PDMS block, which was partially cured for 25 minutes at . The PDMS block was then peeled from the mold and the outlet ports of the micro-valves were fabricated by punching a hole into the PDMS block with a 1 *mm* biopsy punch (Harris Uni-Core, Redding, CA). Simultaneously the PDMS layer on the first mold was partially cured by placing it on a hot-plate for 4 minutes at . The main PDMS block already fabricated which contains the control layer was aligned manually with the flow layer. Both parts which were partially cured were sealed to each-other by completely curing them in an oven at for 15 minutes Then the PDMS block was removed from the mold and the reservoirs were fabricated by cutting along a photo-lithographically defined rectangle of calculated dimensions, and a 4 *mm* biopsy punch (Harris Uni-Core, Redding, CA) for the focused stream. A 60×22 *mm* borosilicate #1 microscope cover-glass (Menzel-Gläser) was treated with BSA (Bovine Serum Albumin) in order to avoid specific adhesion of platelets to the cover-slide as follows: BSA was placed onto the cover-slide and left for 1h, then the cover-slide was rinsed with DI water and dried with pressurized nitrogen. Finally, the fabricated PDMS chip was surface adhered to the BSA treated microscope cover-glass which formed the bottom wall of the micro-channel and allowed for both transmitted light and epi-fluorescence imaging at the micro-contraction geometries.

### Equations used for designing the microfluidic flow focusing device

**Length of the inlet feeders** Given the initial length of the core stream as an input, the lengths of the resistances as a function of the desired widths (core and focused stream) were derived as:

(2)

where γ is the ratio of average velocities of main channel and focused channel i.e. .

**Reservoir size.** In order to maintain the thickness of the stream constant during the whole experiment, the pressure drop of the core and focused channel from the reservoir to the intersection, should be equivalent (i.e ). This was achieved by designing the reservoirs such that the changes in the hydrostatic pressure were the same for both (Δ*P*=ρ*gH*), which means that the height of the column of the liquid in both reservoirs should remain identic during the whole experiment (, where ), this implies that , for a two stream device and , for a three stream device, where , and are the area of the reservoirs. For a steady-state flow, the pressure across a channel was assumed constant.

### Vortex formation as a function of platelet aggregate size. See Video.

Video S1
